# Acute Myocardial Infarction in an Intermediate-Risk Patient: Implications for Cardiovascular Risk Stratification

**DOI:** 10.1016/j.jaccas.2025.106590

**Published:** 2026-01-21

**Authors:** Oludamilola Akinmolayemi, Devarshi Vasa, Deepak L. Bhatt

**Affiliations:** a Mount Sinai Fuster Heart Hospital, Icahn School of Medicine at Mount Sinai, New York, New York, USA; b Icahn School of Medicine at Mount Sinai, New York, New York, USA

**Keywords:** acute myocardial infarction, cardiovascular risk stratification, coronary artery calcium score, coronary artery disease, primary prevention

## Abstract

**Background:**

Cardiovascular risk stratification is essential and informative for long-term prognostication. However, it is often limited by heterogeneous patient profiles.

**Case Summary:**

An asymptomatic 76-year-old man with a history of multiple myeloma in remission presented for cardiovascular risk assessment. Despite intermediate-risk classification of atherosclerotic cardiovascular disease using risk stratification scores, the coronary artery calcium score (73 Agatston units) was used to guide decision-making regarding initiation of lipid-lowering therapy. However, before therapy initiation, the patient experienced an ST-segment elevation myocardial infarction.

**Discussion:**

Cardiovascular risk stratification tools serve an important role in evaluating patient risk and facilitating shared decision-making in clinical management. Nonetheless, these tools present certain limitations in predicting comprehensive risk outcomes, especially when used independently.

**Key Takeaways:**

This case highlights the intricacies associated with cardiovascular risk stratification tools and reinforces the importance of using a multimodal assessment strategy to inform the initiation of preventive therapies.

## Visual Summary

**Figure:**
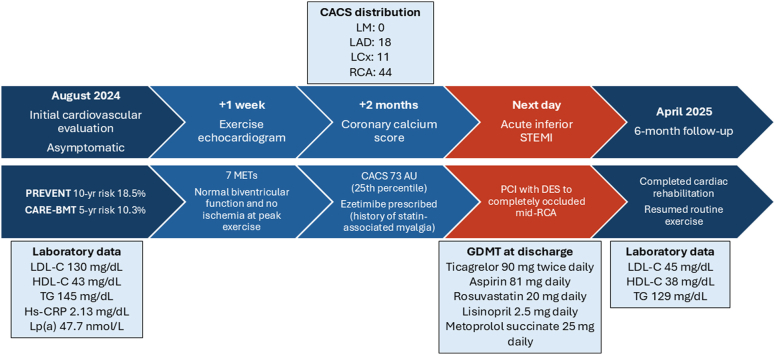

## History of presentation

**Figure 1:**
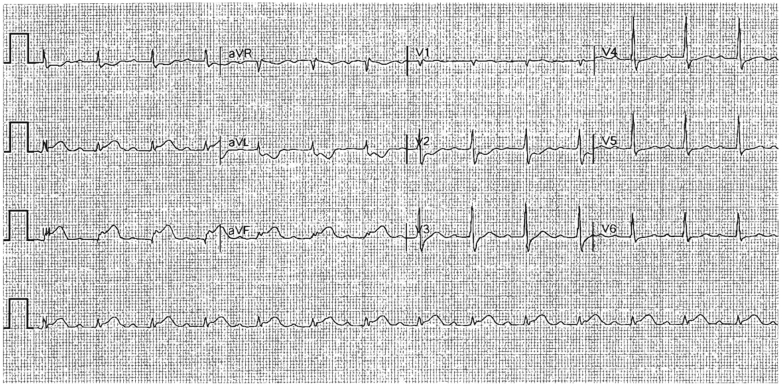
Electrocardiogram on Presentation to the Outside Hospital Emergency Department ST-segment elevation was noted in the inferior leads (II, III, and aVF) with prominent reciprocal ST-segment depression in the lateral leads (I and aVL), consistent with an acute inferior STEMI. STEMI = ST-segment elevation myocardial infarction.

A 76-year-old man reported a sensation of heaviness in the left upper chest radiating to the right chest, which he initially considered to be gastric reflux. The symptoms persisted and eventually resulted in syncope. Emergency medical services were contacted; the patient was found to have a high-degree atrioventricular block (2:1 atrioventricular block vs complete heart block) with ventricular rates in the 30s and inferior wall myocardial infarction on electrocardiogram. Emergency medical services administered 1 mg atropine, leading to conversion to sinus rhythm. The patient was transported to the nearest emergency department, where inferior wall myocardial infarction was confirmed on electrocardiogram (Figure 1).

**Take-Home Messages**

• Traditional cardiovascular risk equations help guide long-term risk assessment, but they do not capture near-term vulnerability to acute coronary syndromes.
• Multimodal cardiovascular risk assessment, proactive patient engagement, and early preventive therapy remain essential to avoid preventable acute cardiovascular events.

## Past medical history

The patient had a history of multiple myeloma and had received autologous hematopoietic stem cell transplantation (HSCT), which was complicated by a prolonged hospital admission. He remained in remission and on maintenance monoclonal antibody therapy with daratumumab. He had a prior diagnosis of hypercholesterolemia, and lipid-lowering therapy was discontinued by the patient owing to concerns about side effects such as myalgias. No family history of cardiovascular disease was reported.

**Visual Summary:**
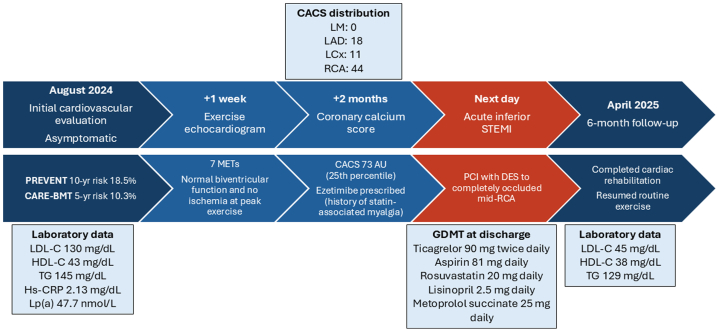
Timeline of Initial Cardiovascular Evaluation, Acute Coronary Event, and Most Recent Follow-Up CACS = coronary artery calcium score; CARE-BMT = Cardiovascular Registry in Bone Marrow Transplantation; DES = drug-eluting stent; GDMT = guideline-directed medical therapy; HDL-C = high-density lipoprotein cholesterol; Hs-CRP = high-sensitivity C-reactive protein; LAD = left anterior descending artery; LCx = left circumflex artery; LDL-C = low-density lipoprotein cholesterol; LM = left main artery; Lp(a) = lipoprotein (a); MET = metabolic equivalent; PCI = percutaneous coronary intervention; PREVENT = Predicting Risk of Cardiovascular Disease EVENTs; RCA = right coronary artery; STEMI = ST-segment elevation myocardial infarction; TG = triglycerides.

## Differential diagnosis

The differential diagnosis of the etiology of the acute myocardial infarction (AMI) in this patient included coronary artery plaque rupture with thrombosis, coronary artery dissection, coronary vasospasm, and embolism.

## Investigations

About 2 months before the AMI, the patient presented to our clinic for a routine cardiovascular assessment. He reported no cardiac symptoms, adhered to a healthy diet, and engaged in moderate-intensity exercise. Notably, before his diagnosis of multiple myeloma, the patient had been an avid cyclist for over 25 years; however, he had not resumed his pretreatment activity levels, which prompted this outpatient evaluation below, prior to the myocardial infarction mentioned above (Visual Summary).

Vital signs were within normal limits, with a blood pressure of 112/73 mm Hg and a heart rate of 65 beats/min. Physical examination was unremarkable.

Initial laboratory evaluation revealed a lipid profile with low-density lipoprotein cholesterol at 130 mg/dL, high-density lipoprotein cholesterol at 43 mg/dL, and triglycerides at 145 mg/dL. High-sensitivity C-reactive protein was mildly elevated to 2.13 mg/dL, while lipoprotein(a) was within normal limits at 47.7 nmol/L. The PREVENT (Predicting Risk of Cardiovascular Disease EVENTs) score estimated the 10-year and 30-year total cardiovascular disease risks at 18.5% and 36.3%, respectively, categorizing the patient as intermediate risk. Considering his history of HSCT, the CARE-BMT (Cardiovascular Registry in Bone Marrow Transplantation) risk score, which is used in patients undergoing HSCT, was calculated and predicated 1-year and 5-year cardiovascular event incidences of 4.0% and 10.3%, respectively. Given the patient being older than 75 years, use of the Atherosclerotic Cardiovascular Disease (ASCVD) 10-year risk calculator was deferred.

In light of the patient's concerns regarding the safety of resuming structured exercise, despite a lack of symptoms and not being directly supported by current guidelines,^1^ an exercise echocardiogram was conducted. The patient achieved 7 metabolic equivalents, placing him in the 50th to 74th percentile for his age and sex,^2^ without evidence of myocardial ischemia at peak exertion. Two months later, given the patient's intermediate risk profile, absence of risk enhancers, and hesitancy to initiate lipid-lowering therapy, a coronary artery calcium score (CACS) was obtained. The results demonstrated a total calcium score of 73 Agatston units (left main: 0, left anterior descending: 18, left circumflex: 11, right coronary artery: 44), placing the patient in the 25th percentile when adjusted for age, sex, and race/ethnicity.

## Management

**Figure 2:**
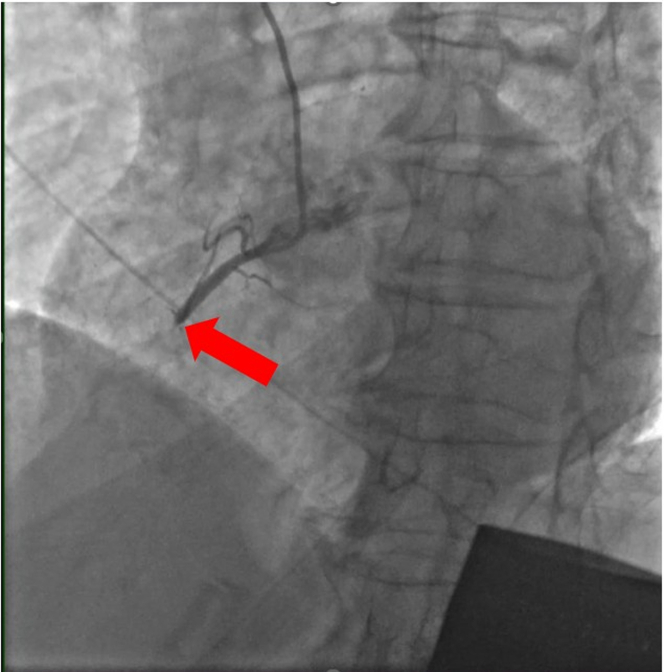
Coronary Angiogram of RCA Before PCI Total occlusion of mid RCA (arrow). PCI = percutaneous coronary intervention; RCA = right coronary artery.

**Figure 3:**
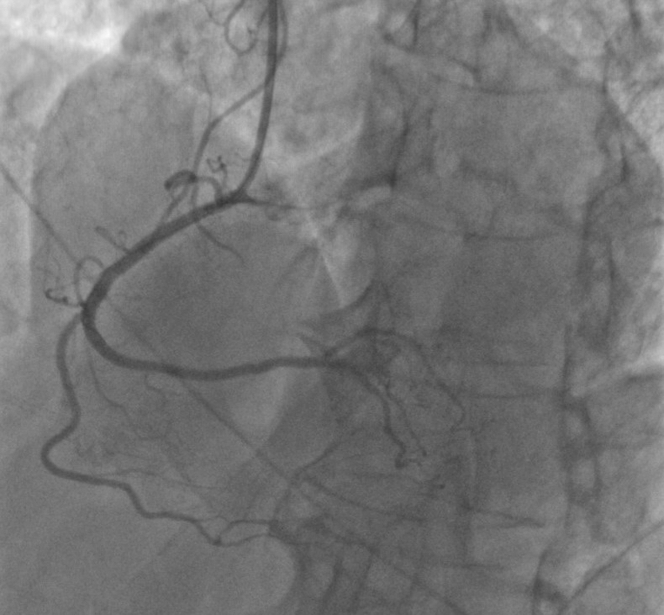
Coronary Angiogram of RCA After PCI With DES TIMI flow grade 3 was achieved. DES = drug-eluting stent; PCI = percutaneous coronary intervention; RCA = right coronary artery.

Ezetimibe therapy was prescribed owing to reported statin-associated myalgia. The day after his coronary artery calcium testing and before starting ezetimibe, the patient experienced the AMI. He underwent emergent percutaneous coronary intervention with drug-eluting stent implantation, which successfully revascularized a completely occluded mid right coronary artery (Figures 2 and 3). He was admitted to the intensive care unit for postcatheterization monitoring and was subsequently discharged on guideline-directed medical therapy, including rosuvastatin 20 mg daily, ticagrelor 90 mg twice daily, aspirin 81 mg daily, lisinopril 2.5 mg daily, and metoprolol succinate 25 mg daily.

## Outcome and follow-up

**Figure 4:**
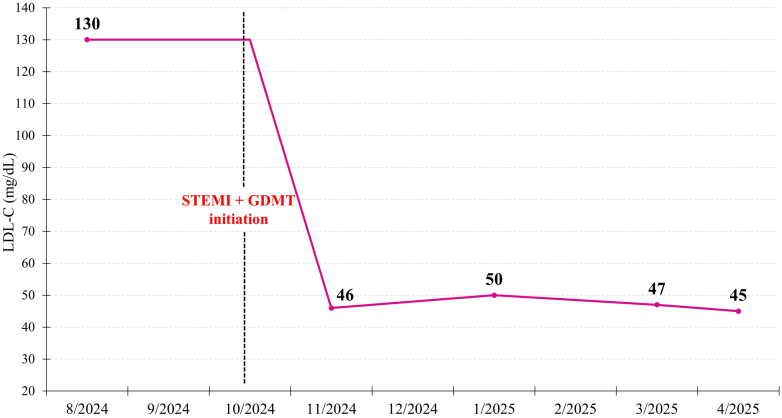
Trend in LDL-C Levels From Initial Evaluation in August 2024 to Most Recent Follow-Up in April 2025 A significant reduction was observed after STEMI presentation in October 2024 and after initiation of GDMT (rosuvastatin 20 mg daily, ticagrelor 90 mg twice daily, aspirin 81 mg daily, lisinopril 2.5 mg daily, and metoprolol succinate 25 mg daily). GDMT = guideline-directed medical therapy; LDL-C = low-density lipoprotein; STEMI = ST-segment elevation myocardial infarction.

At follow-up, improvements were observed in lipid parameters, he completed cardiac rehabilitation, and he resumed his exercise regimen (Figure 4).

## Discussion

Cardiovascular disease (CVD) affects approximately 50% of adults in the United States, with prevalence increasing with age across both sexes.^3^ Coronary artery disease accounts for a substantial proportion of CVD-related deaths, and early identification as well as modification of risk factors, along with the prevention of subclinical atherosclerosis progression, are important for primary prevention of coronary artery disease.^3^ Risk assessment tools, including the 2013 ASCVD risk calculator and the more recent PREVENT equation, have been developed to estimate long-term CVD risk. These tools support risk stratification, facilitate shared decision-making between physicians and patients, and inform evidence-based preventive clinical interventions.

This case highlights the inherent limitations of conventional cardiovascular risk assessment tools. In this patient, the PREVENT and CARE-BMT risk scores indicated an intermediate 10-year and 5-year CVD risk, respectively. While these assessments are intended for long-term risk stratification to guide primary prevention management, they do not diagnose or predict imminent cardiovascular events, emphasizing the importance of promptly initiating preventive therapy when elevated risk is identified.

The CACS is increasingly recognized as a valuable adjunctive tool in cardiovascular risk assessment, particularly in patients whose risk profiles fall within intermediate categories and when therapy decisions such as statin initiation are uncertain.^4^ The CACS directly reflects the burden of calcified plaque within the coronary arteries and correlates closely and in a graded association with long-term incident cardiovascular outcomes.^5^ However, as demonstrated in this case, a CACS may underestimate ASCVD risk, as it does not provide information regarding total plaque burden, specifically low-attenuation plaque, which has been shown to strongly and independently predict fatal or nonfatal myocardial infarction.^6^ Recent studies suggest that although CACS enhances traditional cardiovascular risk prediction, it does not provide comprehensive insights into plaque stability or the presence of noncalcified vulnerable lesions.^7^ In our patient, a CACS of 73 Agatston units may have been reassuring in isolation, but this case highlights the need to interpret the CACS in conjunction with other risk factors.

The occurrence of an acute coronary syndrome in our patient despite undergoing comprehensive cardiovascular evaluations highlights the importance of initiating primary prevention strategies, such as lipid-lowering therapy, promptly upon recognition of elevated ASCVD risk. Delays in medication initiation or exclusive dependence on risk stratification may insufficiently allow provision of appropriate care to those at increased risk for cardiovascular events.^8^ Contemporary guidelines stress the necessity of integrating individualized risk assessments with supplementary approaches, including biomarkers and imaging, particularly for patients presenting with distinct clinical risk factors.^9^

This case further underscores the challenges clinicians face when addressing patient reluctance toward pharmacological interventions, particularly in relation to concerns about adverse effects such as statin-induced myalgia. It highlights the imperative of comprehensive patient education, collaborative decision-making, and the judicious consideration of alternative lipid-lowering agents, including ezetimibe, proprotein convertase subtilisin/kexin type 9 (PCSK9) inhibitors, or other nonstatin options when appropriate. Clinicians are encouraged to proactively engage with patients to address their concerns and promote adherence to essential preventive therapies.

## Conclusions

In summary, this case highlights the importance of a comprehensive, multimodal approach to cardiovascular risk assessment tailored to patient comorbidities. Clinicians should use established risk scores, incorporate biomarkers and imaging as needed, and start preventive therapies early. Further research, including the use of coronary computed tomography angiography,^10^ is needed to identify high-risk asymptomatic patients prior to acute ischemic events.

## Funding Support and Author Disclosures

Dr Bhatt discloses the following relationships: Advisory board: Angiowave, Antlia Bioscience, Bayer, Boehringer Ingelheim, CellProthera, Cereno Scientific, E-Star Biotech, High Enroll, Janssen, Level Ex, McKinsey, Medscape Cardiology, Merck, NirvaMed, Novo Nordisk, Repair Biotechnologies, Stasys, SandboxAQ (stock options), Tourmaline Bio; board of directors: American Heart Association New York City, Angiowave (stock options), Bristol Myers Squibb (stock), DRS.LINQ (stock options), High Enroll (stock); consultant: Alnylam, Altimmune, Broadview Ventures, Corcept Therapeutics, Corsera, GlaxoSmithKline, Hims, SERB, SFJ, Summa Therapeutics, Worldwide Clinical Trials; data monitoring committees: Acesion Pharma, Assistance Publique-Hôpitaux de Paris, Baim Institute for Clinical Research, Boston Scientific (chair, PEITHO trial), Cleveland Clinic, Contego Medical (chair, PERFORMANCE 2), Duke Clinical Research Institute, Mayo Clinic, Mount Sinai School of Medicine (for the ABILITY-DM trial, funded by Concept Medical; for ALLAY-HF, funded by Alleviant Medical), Novartis, Population Health Research Institute; Rutgers University (for the NIH-funded MINT Trial); honoraria: American College of Cardiology (senior associate editor, Clinical Trials and News, ACC.org; chair, ACC Accreditation Oversight Committee), Arnold and Porter law firm (work related to Sanofi/Bristol-Myers Squibb clopidogrel litigation), Baim Institute for Clinical Research (AEGIS-II executive committee funded by CSL Behring), Belvoir Publications (editor in chief, Harvard Heart Letter), Canadian Medical and Surgical Knowledge Translation Research Group (clinical trial steering committees), CSL Behring (AHA lecture), Duke Clinical Research Institute, Engage Health Media, HMP Global (editor in chief, Journal of Invasive Cardiology), Medtelligence/ReachMD (CME steering committees), MJH Life Sciences, Oakstone CME (course director, Comprehensive Review of Interventional Cardiology), Philips (Becker's Webinar on AI), Population Health Research Institute, WebMD (CME steering committees), Wiley (steering committee); other: Clinical Cardiology (deputy editor, unpaid); Progress in Cardiovascular Diseases (deputy editor); Added Health (editorial board; stock options); Patent: Sotagliflozin (named on a patent for sotagliflozin assigned to Brigham and Women's Hospital who assigned to Lexicon; neither I nor Brigham and Women's Hospital receive any income from this patent); research funding: Abbott, Acesion Pharma, Afimmune, Alnylam, Amarin, Amgen, AstraZeneca, Atricure, Bayer, Boehringer Ingelheim, Boston Scientific, CellProthera, Cereno Scientific, Chiesi, Cleerly, CSL Behring, Faraday Pharmaceuticals, Fractyl, Idorsia, Janssen, Javelin, Lexicon, Lilly, Medtronic, Merck, MiRUS, Moderna, Novartis, Novo Nordisk, Pfizer, PhaseBio, Regeneron, Reid Hoffman Foundation, Roche, Sanofi, Stasys, 89Bio; Royalties: Elsevier (editor, Braunwald's Heart Disease); site co-investigator: Cleerly. All other authors have reported that they have no relationships relevant to the contents of this paper to disclose.
